# Torsion of Wandering Spleen with Infarction

**DOI:** 10.7759/cureus.3177

**Published:** 2018-08-21

**Authors:** Dawar B Khan, Kumail Khandwala, Summar-un-nisa Abbasi, Sarim D Khan, Rabail Raza

**Affiliations:** 1 Department of Radiology, The Aga Khan University, Karachi, PAK; 2 Medical College, The Aga Khan University, Karachi, PAK

**Keywords:** wandering spleen, torsion, infarction, hilar

## Abstract

Wandering spleen is a rare entity that results from the absence or maldevelopment of the ligaments that support the spleen in its normal location. As a result, the spleen is hypermobile and may be predisposed to hilar torsion and subsequent infarction, making it a potentially fatal abdominal emergency. We present a case of a 36-year-old Afghan female who presented with an acute abdomen, and was radiologically and surgically confirmed to have a wandering spleen with torsion and complete infarction. Knowledge of this condition and its radiological findings can play a crucial role in making a correct and timely diagnosis.

## Introduction

Wandering spleen is a rare clinical entity that results from the absence or maldevelopment of the ligaments that support the spleen in its normal location [1]. As a result, the spleen is hypermobile and may be predisposed to hilar torsion and subsequently infarction, making it a potentially fatal emergency. It is an uncommonly encountered condition that mainly affects the pediatric population in one third of cases [2]. In adults, females of reproductive age group are mostly affected, with the cause hypothesized to be hormonal changes during pregnancy leading to ligamentous laxity [2,3]. We present a case of a 36-year-old female who presented with this condition. Knowledge of this entity and its radiological findings can play a crucial role in making a correct and timely diagnosis.

## Case presentation

A 36-year-old multiparous Afghan woman presented to the emergency department with a history of abdominal pain and vomiting for one week with sudden increase in intensity of pain for the last three hours. There was no history of fever, prior surgeries or trauma. On physical examination, abdominal distension was noted. There was diffuse abdominal tenderness more pronounced in the lower abdomen, but no definite palpable mass was felt.

**Figure 1:**
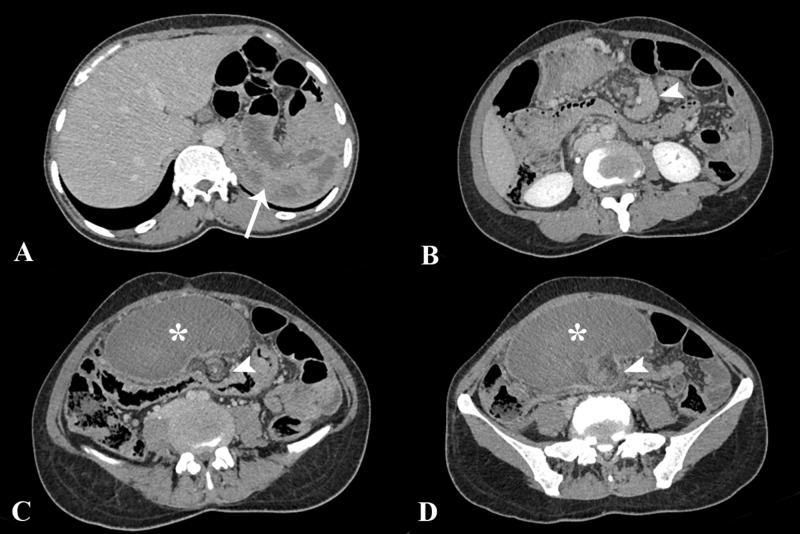
Contrast-enhanced computed tomography images. Axial sections showing absence of spleen in its normal position in the left upper quadrant (arrow). An enlarged spleen (asterisk) was found located in the lower abdomen, reaching up to the pelvis. Low attenuation of the splenic parenchyma was noted with elongated and twisted vascular pedicle (arrowheads). The pancreatic tail was also involved in the twisting of the pedicle as seen in B (arrowhead).

**Figure 2:**
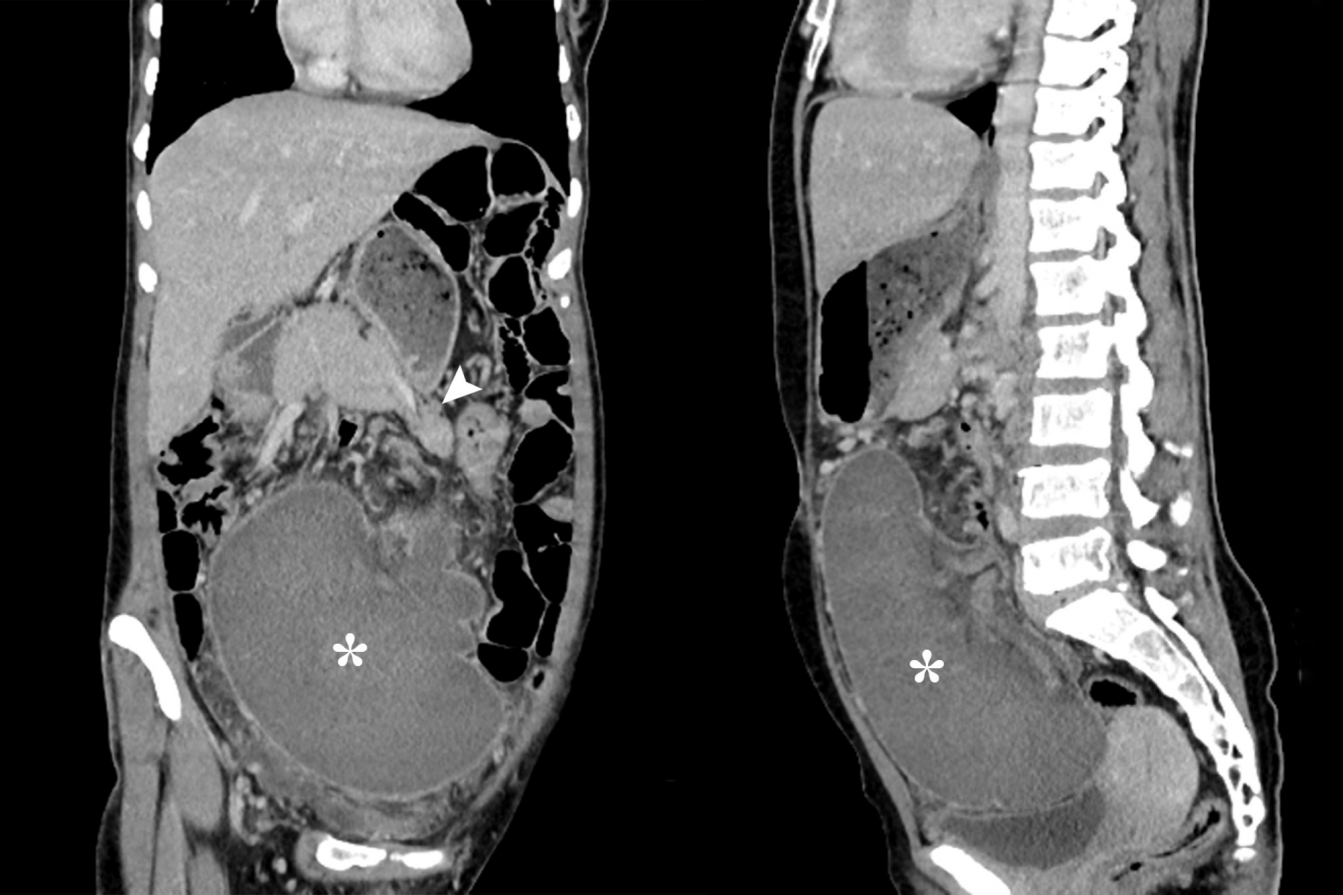
Coronal and sagittal computed tomography images. Reformatted images showing abnormal position of the spleen (asterisk). Also seen is the pancreatic tail being pulled along with the vascular pedicle (arrowhead).

Computed tomography (CT) was performed which revealed absence of the spleen in the left upper quadrant. An abnormally placed, enlarged spleen was noted in the lower abdomen reaching up to the pelvis. It had an elongated and twisted vascular pedicle. On contrast-enhanced images there was a lack of parenchymal enhancement in the spleen with homogenous low attenuation and peripheral enhancement. The pancreatic tail was also involved in the torted pedicle (Figures 1, 2).

**Figure 3:**
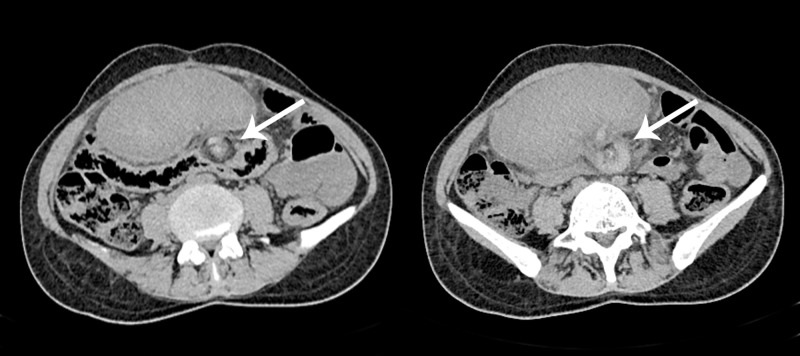
Non-contrast computed tomography images. Axial sections from the abdomen and pelvis showing hyperdense splenic pedicle and characteristic whorled appearance of the splenic vessels and fat, representative of torsion of wandering spleen (arrows).

Non-contrast images confirmed the typical findings of an abnormally located spleen, with a hyperdense splenic pedicle and whorling of the vessels and fat, which were characteristic of torsion (Figure 3).

The findings were concluded as wandering spleen with hilar torsion and liquefactive infarction. The patient underwent an exploratory laparotomy. On surgery, the spleen appeared congested and infarcted, and the splenic vessels were thrombosed. Therefore, total splenectomy was performed because of non-viability. The post-operative recovery of the patient was uneventful and she was discharged after two weeks.

## Discussion

Wandering spleen is a rare entity in which the spleen is hypermobile and ectopic in position, and attached only by an elongated vascular pedicle which becomes prone to torsion. Wandering spleen is found in less than 0.5% of splenectomies and occurs mainly in children and women aged 20-40 years [4]. This condition is a result of congenital anomalies in the development of the dorsal mesogastrium and the absence or maldevelopment of normal splenic suspensory ligaments resulting in laxity. The splenic ligaments include the gastrolienal and lienorenal ligaments which attach the spleen to the stomach and posterior abdominal wall respectively, and the phrenicocolic ligament which supports the spleen inferiorly [1,5].

Acquired anomalies have also been described to cause laxity of the supporting ligaments. These may be secondary to abdominal wall weakness, multiple pregnancies, and hormonal changes especially in reproductive females [3]. Conditions that cause an increase in size of the spleen, like lymphoma, chronic myeloid leukemia and malaria have also been implicated in the etiology [5]. Both congenital and acquired conditions result in an elongated pedicle, which is predisposed to torsion and may result in partial or complete infarction because the splenic vessels course within it.

Clinical symptomatology is variable and may range from the patient being completely asymptomatic, presenting with a mobile abdominal lump, or having intermittent abdominal pain because of partial torsion and spontaneous detorsion of the splenic pedicle. Often, patients may also present with an acute abdomen due to complete torsion and infarction. On examination, a firm, mobile abdominal mass with characteristic “notched borders” may be felt, but this is always not the case because splenic engorgement may obliterate the splenic notch and therefore a clinical diagnosis is usually tricky [3-5].

Radiology plays a crucial role in reaching the correct diagnosis. Plain radiography and barium studies are often non-specific and findings may include non-visualization of the splenic shadow or visualization of gas-filled bowel loops in the left upper quadrant (LUQ). A large central or lower abdominal soft tissue mass may be seen, and on barium studies there may be displacement of splenic flexure with extrinsic impression by the mass [6]. On sonography, the spleen is not present in the splenic bed in the LUQ, and instead a capsulated mass is visualized in the abdomen or pelvis. This mass may demonstrate a heterogenous or hypoechoic echotexture with reduced or absent intraparenchymal and hilar color flow on Doppler, depending on the degree of torsion [7]. Angiography can also show an abruptly twisted distal splenic artery at the point of torsion, but it is invasive and therefore not used for diagnostic purposes [1]. In addition, splenic scintigraphy with heat-damaged red blood cells, which has its applications in detecting splenic tissue in cases of splenosis (accessory spleens) and ectopic spleens, has also been used previously for confirming the diagnosis in ambiguous cases [8].

Computed tomography (CT), however, is the modality of choice for diagnosing a wandering spleen when torsion is suspected. CT findings include ectopic position of the spleen, whorled appearance of the splenic pedicle and surrounding fat at the splenic hilum, which may or may not be accompanied by twisting of the pancreatic tail [8,9]. The spleen may be enlarged due to hypercongestion and there may be minimal or absent post-contrast enhancement of the splenic parenchyma which is usually a sign of infarction [8,10]. Non-contrast CT may reveal lower attenuation values of the spleen in comparison to the hepatic parenchyma and a hyperdense splenic pedicle may be seen, which is indicative of the thrombosed splenic vein [10]. Secondary findings such as ascites may also be noted. Magnetic resonance imaging (MRI) can help in the diagnosis as well, showing characteristic signals of infarction on both T1- and T2-weighted images [11] and loss of signal void of the splenic pedicle vessels suggesting thrombosis. Rare complications like gastric, pancreatic tail or splenic flexure volvulus and dolichosigmoid of the colon have also been reported previously in association with wandering spleen [1,5,12,13].

Our case showed typical CT findings of a wandering spleen with torsion of its pedicle and complete liquefactive infarction. The findings were confirmed during surgery and subsequent histopathological examination. Until recently, splenectomy has been done for wandering spleen although many surgeons now recommend open or laparoscopic splenopexy for viable cases. Therefore, partial or total splenectomy should be performed in patients with splenic torsion, in whom infarction and thrombosis of the splenic vessels has occurred [14,15].

## Conclusions

Torsion and infarction of a wandering spleen is a rare abdominal emergency. Characteristic imaging features on radiological modalities like Doppler and CT scan are crucial in making an accurate and timely diagnosis. A fair degree of accurate assessment about viability and thrombosis of the splenic vessels can be made on CT scan which may help the surgeon decide the right mode of treatment.
